# Opinion: Inhibition of Blood-Brain Barrier Repair as a Mechanism in HIV-1 Disease

**DOI:** 10.3389/fnins.2017.00228

**Published:** 2017-04-26

**Authors:** Monique E. Maubert, Brian Wigdahl, Michael R. Nonnemacher

**Affiliations:** ^1^Department of Microbiology and Immunology, and Center for Molecular Virology and Translational Neuroscience, Institute for Molecular Medicine and Infectious Disease, Drexel University College of MedicinePhiladelphia, PA, USA; ^2^Sidney Kimmel Cancer Center, Thomas Jefferson UniversityPhiladelphia, PA, USA

**Keywords:** HIV-1, Tat, blood-brain barrier (BBB), NMDAR, VEGFR, c-Src

The blood-brain barrier (BBB) is a complex network of microvasculature, comprised primarily of brain microvascular endothelial cells (BMECs), astrocytes, and pericytes, which regulates cellular, macromolecule, and metabolite passage between the peripheral circulation and the central nervous system (CNS). Damage to the BBB has been linked to neurocognitive deficits sustained in multiple diseases, including stroke, Alzheimer's Disease, and numerous infections, including human immunodeficiency virus type 1 (HIV-1) (Krizanac-Bengez et al., 2004; Salmina et al., 2010; Logsdon et al., 2015). Although the development and deployment of anti-retroviral therapy (ART) has transformed HIV-1 infection from an acute terminal diagnosis to a chronic pharmaceutically-managed clinical condition (in the developed world), many clinical complications remain prevalent in HIV-1-infected patients, including the spectrum of neurocognitive deficits collectively termed HIV-1-associated neurocognitive disorders (HAND). While the current age of ART has decreased the occurrence of the more severe manifestations of neurocognitive impairment in patients, particularly with regards to the incidence of HIV-1-associated dementia (HAD), the overall prevalence of HAND has not subsided (Cysique et al., 2004; Robertson et al., 2007; Tozzi et al., 2007; Heaton et al., 2010, 2011; Cysique and Brew, 2011). In fact, it is currently estimated that nearly 50–70% of HIV-1-infected patients on a successful ART regimen experience some level of neurocognitive decline (Heaton et al., 2010, 2011, 2015; Simioni et al., 2010; Obermeit et al., 2017). Implicated in the development of HAND in patients is a combination of toxic viral proteins released into the CNS, a sustained host pro-inflammatory response in the CNS initiated by the virus, deregulated endogenous small molecule metabolism, detrimental metabolic byproducts associated with combination ART, as well as certain types of HIV-1 genetic variants that may cause some of these pathogenic processes (Krebs et al., 2000; Wang et al., 2006; Ferrucci et al., 2011, 2012; Strazza et al., 2011; Dahiya et al., 2013; Aiamkitsumrit et al., 2014; Gresele et al., 2014; Maubert et al., 2015; Dampier et al., 2017; James et al., 2016).

Numerous *in vitro* and *in vivo* models have demonstrated molecular deregulation and functional impairment of the BBB in the context of HIV-1 infection, including downregulation of key tight junction complex components and upregulation of cell adhesion molecules on BMECs, aberrant activation of astrocytes and pericytes, overall increased permeability, and enhanced immune cell passage across the barrier (Toborek et al., 2005; Li et al., 2009; Roberts et al., 2010; Debaisieux et al., 2012; Louboutin and Strayer, 2012; Nakagawa et al., 2012; Bagashev and Sawaya, 2013; Niu et al., 2014; Rao et al., 2014; Hong and Banks, 2015; Maubert et al., 2015). However, while multiple HIV-1 proteins (including Tat, gp120, Vpr, and Nef) have been shown to deregulate numerous pathways resulting in *damage* to the BBB, the role of HIV-1 in modulating the pathways relative to the *repair* of the BBB and re-endothelialization (or the inhibition, thereof) have not yet been reported in detail.

Clinically, endothelial cell dysfunction and general wound repair has been problematic in the HIV-1-infected patient population, with several reports highlighting delays in healing time and related complications, including secondary infections of these wounds (Lord, 1997; Davis et al., 1999; Diz Dios et al., 1999; Miyamoto et al., 2006; Arildsen et al., 2013; Wang et al., 2013; Francisci et al., 2014; Balsam et al., 2015; Fitzpatrick et al., 2016). While the mechanisms orchestrating endothelial repair, particularly considering those of the BBB, are not entirely defined, some molecular effectors and interconnecting pathways have been identified in the literature for their demonstrated involvement in repair processes in various model systems. In particular, cellular Src kinase (c-Src), a ubiquitously expressed member of the Src family of non-receptor tyrosine kinases, has a defined role in endothelial cell regulation and repair, both *in vitro* and *in vivo* (Takenaga et al., 2009; Liu et al., 2010; Franco et al., 2013; Bai et al., 2014; Cao et al., 2015), the specifics of which are described below. Importantly, c-Src is involved in pathways linked to the activation of vascular endothelial growth factor receptor 2 (VEGFR2) and N-methyl D-aspartate receptor (NMDAR), both of which are expressed on human BMECs and with demonstrated roles in BBB regulation and integrity (Sharp et al., 2003, 2005; Andras et al., 2007; Holmes et al., 2007; Davis et al., 2010; Reijerkerk et al., 2010; Hudson et al., 2014; Chen et al., 2016; Fearnley et al., 2016).

Of note, HIV-1 infection and c-Src activation have been shown to have a reciprocal relationship in the literature with reports demonstrating that c-Src activation was increased in human Jurkat T cells 24 h after HIV-1 infection (Phipps et al., 1996) and in activated primary human CD4 T cells within 1 h of infection, as compared to uninfected controls, and that both chemical inhibition and siRNA knockdown of c-Src decreased infectivity of Nef-deficient HIV-1 reporter viruses by more than 50% in primary human CD4 T cells, *in vitro* (McCarthy et al., 2016). In addition, siRNA knockdown of c-Src decreased proviral integration of Nef-competent X4 and R5 HIV-1 laboratory strains by several-fold and significantly attenuated replication of these viral strains in primary human CD4 T cells, *in vitro* (McCarthy et al., 2016). While this observation links c-Src to HIV-1 infection in cells, it is known that BMECs and endothelial cells in general are not infected.

Given this point, it is more likely that extracellular viral proteins would interact with the BMECs causing dysfunction or inhibiting repair. At the level of isolated viral proteins, previous reports have indicated that HIV-1 gp120 can activate the NMDAR through direct binding of NMDAR subunits (Xin et al., 1999) in numerous *in vitro* systems, including in primary rat (Pattarini et al., 1998) and human (Pittaluga et al., 1996) neuronal synaptosomes; indirect activation of the NMDAR by gp120 exposure through the enhanced secretion of NMDAR agonists from proximal glia (Meucci and Miller, 1996) as well as activation of other receptor-mediated pathways which affect NMDAR activity in primary rat cultures (Xu et al., 2011) has also been reported. Additionally, it has been demonstrated that HIV-1 Nef activates c-Src in an *in vitro* yeast model system (Trible et al., 2006; Narute and Smithgall, 2012). Most strikingly, though, c-Src, VEGFR2, and NMDAR have all been shown to be activated by exposure to HIV-1 Tat protein in a number of cell types, suggesting that Tat may be involved in inhibiting the mechanisms of BBB repair in HIV-1 disease. With respect to Tat, this may be of particular importance in ART-suppressed patients given recent evidence that shows Tat can be detected in cells, cerebral spinal fluid, and plasma of these individuals (Falkensammer et al., 2007; Mediouni et al., 2012; Bachani et al., 2013).

To further support the role of c-Src in BBB health and repair, it has been reported that inhibition of c-Src by siRNA limited permeability of human umbilical vein endothelial cells (HUVEC) exposed to vascular endothelial growth factor (VEGF, a known inducer of permeability of the BBB; Holmes et al., 2007; Davis et al., 2010; Hudson et al., 2014; Cao et al., 2015; Fearnley et al., 2016). In addition, chemical inhibition of c-Src with the inhibitor 1-(1,1-dimethylethyl)-3-(4-methylphenyl)-1H-pyrazolo[3,4-d]pyrimidin-4-amine (PP1) accelerated healing of wounded HUVEC (Franco et al., 2013), *in vitro*. Furthermore, *in vivo* treatment with the broad Src family inhibitor 4-amino-5-(4-chlorophenyl)-7-(t-butyl)-pyrazolo[3,4-d]pyrimidine (PP2) in rats subjected to ischemic insult resulted in the rescue of ischemic BBB leakage (Takenaga et al., 2009) and improved neurological deficit scores (Bai et al., 2014) in the presence of the inhibitor. Moreover, c-Src has been identified as an upstream regulator of a number of tight junction complex components, including occludin (Takenaga et al., 2009), claudin-5 (Bai et al., 2014), and zona occludens-1 (Morin-Brureau et al., 2011), as well as a modulator of NMDAR activity in neurons (Lu et al., 1999; Yu and Salter, 1999; Rong et al., 2001; Heidinger et al., 2002; Hossain et al., 2012; Tang et al., 2012; Krogh et al., 2014), and a downstream effector of the VEGFR2 signaling pathway (He et al., 1999; Morin-Brureau et al., 2011; Sun et al., 2012; Cao et al., 2015), in addition to its role in cell cycle regulation and proliferation (Boggon and Eck, 2004; Parsons and Parsons, 2004; Hu et al., 2008; Sen and Johnson, 2011; Reinecke and Caplan, 2014).

Structurally, c-Src is comprised of several domains, including a myristoylated membrane-targeting SH4 domain at the N-terminus, followed by a unique domain, a SH3 domain, a SH2 domain, a kinase-linker region, a SH1 kinase domain bearing the activating tyrosine site (Y416), and a C-terminus bearing the inhibiting tyrosine site (Y529) (Boggon and Eck, 2004; Parsons and Parsons, 2004; Reinecke and Caplan, 2014). It has been previously reported that protein binding of the SH3 domain orchestrates the physical shift necessary to induce the active conformation of c-Src (Alexandropoulos and Baltimore, 1996). Interestingly, it has previously been shown that HIV-1 Tat binds SH3 domains (Rom et al., 2011), and additional results have demonstrated that Tat exposure activates c-Src in primary rat neurons (Krogh et al., 2014), in a fetal bovine aortic endothelial cell line (Urbinati et al., 2005), and in human renal endothelial cells in the presence of growth factors (Das et al., 2016), supporting the hypothesis that HIV-1 Tat may delay BBB repair through the activation of c-Src in human BMECs.

In addition, characterization of the ubiquitously-expressed c-Src promotor revealed several consensus Sp1 transcription start sites (Bonham and Fujita, 1993) and further analysis confirmed that transcriptional regulation of c-Src is dependent on Sp1 activity at the promotor (Ritchie et al., 2000). The relationship of Tat with Sp1 in the transcriptional regulation of the HIV-1 LTR, in addition to the regulation of several host genes, has been extensively explored in the literature (Harrich et al., 1989; Jeang et al., 1993; Majello et al., 1994; Lim and Garzino-Demo, 2000; Burnett et al., 2009; Miller-Jensen et al., 2013; Kukkonen et al., 2014). In addition, it has been reported that Tat promotes Sp1 phosphorylation and activity and that this is orchestrated by Tat in a DNA-PK (double-stranded DNA-dependent protein kinase)-dependent manner (Chun et al., 1998). These reports altogether suggest that Tat may also influence expression of c-Src at the transcriptional level by direct modulation of Sp1 activity at the c-Src promotor.

Previous reports have demonstrated that cytosolic c-Src localizes primarily with membrane-bound structures (Sen and Johnson, 2011; Reinecke and Caplan, 2014), and is documented to associate via adaptor proteins at the plasma membrane with both VEGFR2 (Holmes et al., 2007; Sun et al., 2012) and NMDAR (Yu and Salter, 1999; Rong et al., 2001; Hossain et al., 2012). VEGFR2 is a transmembrane receptor tyrosine kinase expressed primarily on vascular endothelial cells, including BMECs of the BBB, and is activated by several identified ligands collectively termed VEGFs (Holmes et al., 2007; Zhang et al., 2013; Fearnley et al., 2016). In general, activation of VEGFR2 has been shown to induce leakiness of the BBB, both *in vivo* and *in vitro* (Davis et al., 2010; Hudson et al., 2014). HIV-1 Tat-induced activation of VEGFR2 and related endothelial compromise has also been demonstrated (Albini et al., 1996; Ganju et al., 1998; Mitola et al., 2000; Arese et al., 2001; Andras et al., 2005). Of note, both *in vivo* and *in vitro*, VEGFR2 activation has been linked with activation of c-Src in numerous endothelial cell types (He et al., 1999; Morin-Brureau et al., 2011; Sun et al., 2012; Cao et al., 2015), and Tat exposure has been shown to mediate VEGFR2-initiated activation of c-Src in endothelial cells (Urbinati et al., 2005; Das et al., 2016). Taken together, these reports have strongly suggested a causal link between Tat exposure, VEGFR2 activation, and c-Src activation in endothelial cells of the BBB, which may potentially inhibit BBB repair in patients, which has not yet been reported.

NMDAR is a transmembrane ionotropic glutamate receptor highly expressed in the CNS and characterized primarily in neurons (Kopke et al., 1993; Lee et al., 2014). Recently, expression of functional NMDAR on BMECs has been identified and a demonstration of NMDAR activation resulting in BBB compromise has been made *in vitro* (Sharp et al., 2003, 2005; Andras et al., 2007; Reijerkerk et al., 2010; Chen et al., 2016). Notably, NMDAR activation is itself regulated by c-Src phosphorylation of the NR2 subunits of the NMDAR (Yu and Salter, 1999; Rong et al., 2001; Hossain et al., 2012; Tang et al., 2012), and exogenous Tat-induced activation of NMDAR in rat neurons, *in vitro*, has been reported (Haughey et al., 2001; Song et al., 2003; Krogh et al., 2014). This includes a report that showed Tat-mediated activation of NMDAR in neurons is through Tat activation of c-Src (Krogh et al., 2014). In addition to this, a reciprocal activation between NMDAR and c-Src has been demonstrated in primary rat neuronal cultures. This works through activation of NMDAR which causes a Ca^2+^ influx that activates nitric oxide synthase (NOS) and thus generates nitric oxide (NO). This leads to an induction of the S-nitrosylation of c-Src, which then promotes the auto-phosphorylation of c-Src at the activating tyrosine (Y416), which further phosphorylates the NR2A subunit of NMDAR (Tang et al., 2012). All of this is additionally complicated by a report which demonstrated HIV-1 Tat subtype-specific toxicity of primary rat neurons and NMDAR-expressing HEK cells, wherein markedly decreased cell survival was observed in cultures exposed to recombinant subtype B Tat as compared to subtype C Tat (Li et al., 2008). These observations were attributed to the cysteine to serine variation at position 31 of Tat that has been previously documented between these subtypes, and it was determined that this amino acid difference influenced the ability of Tat to interact with and activate the NMDAR (Li et al., 2008). Taken together, these reports imply an important relationship between HIV-1 Tat exposure, Tat genetics, c-Src activation, and NMDAR activation in endothelial cells of the BBB, which may hinder BBB repair mechanisms, which has not previously been reported.

In summary, as a dynamic biological structure tasked with mediating communication between the CNS and the peripheral circulation, proper regulation of the BBB is critical to the maintenance of homeostasis in the CNS, and perturbations of the mechanisms in place which maintain this dynamic regulation are implicated in the incidence of a number of neurological diseases in humans, including HAND. The literature provides evidence that demonstrates that BBB damage does indeed occur in HIV-1 CNS disease, and that this damage correlates to the spectrum of HAND outcomes reported in patients, however, whether these issues are compounded by a mechanistic inhibition of BBB repair in these patients, has not yet been documented. In addition, it is unclear as to whether the nature of the damage, the initiator of the damage, and whether the precise location or compartment where the damage has occurred are of distinct importance in the context of inhibition of the BBB repair mechanism proposed herein. Given these observations, understanding how extracellular HIV-1 proteins, HIV-1-infected cells, and/or how support cells of the BBB (i.e., astrocytes, pericytes) alter the repair of the BBB and whether the regulation of c-Src is at the center of this question is an open area of significant research to understanding the mechanisms that underlie HIV-1 neuropathogenesis and HAND (Figure 1).

**Figure 1:**
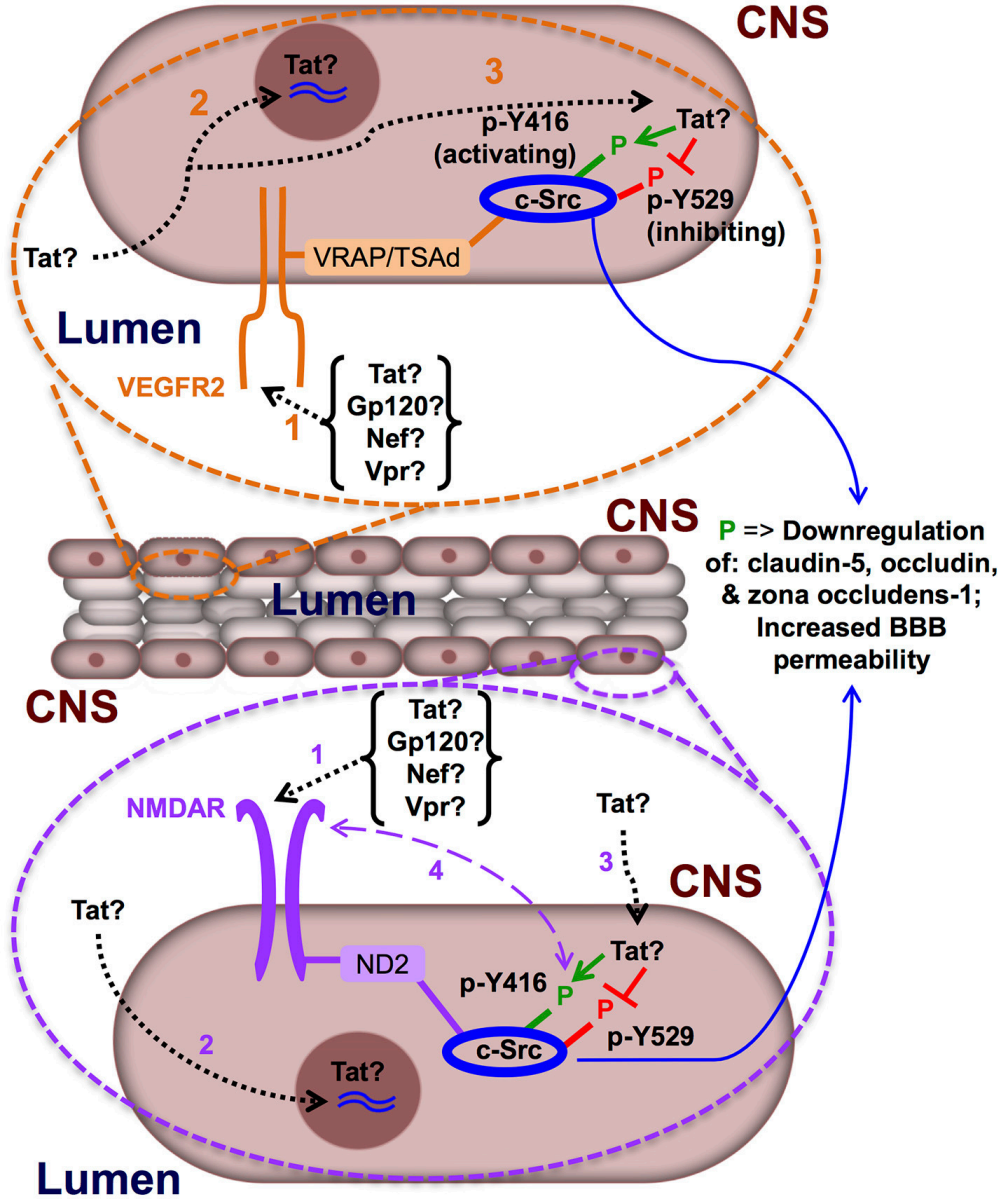
**Hypothetical model of the mechanisms underlying delayed blood-brain barrier repair in HIV-1 infection**. Differential expression of VEGFR2 has been proposed and demonstrated on the lumen- and CNS-exposed surfaces of primary rat BMECs, *in vitro* (Hudson et al., 2014), lending to the possibility that this receptor may be activated by HIV-1 proteins flowing in the peripheral circulation, as well as those generated in the CNS; here, we illustrate expression of VEGFR2 on the lumen-exposed surface (orange bubble). Polarized expression of NMDAR on BMECs has not been addressed in the literature; however, in consideration that the ligands which would activate this receptor are readily found in the CNS and secreted by astrocytes (a component of the BBB in adjacent proximity with BMECs), we presume and illustrate here expression of NMDAR on the CNS-exposed surface (purple bubble). (1) HIV-1 proteins (Tat, gp120, Vpr, or Nef) bind and activate the VEGFR2 and/or NMDAR, stimulating the receptor(s) and resulting in activation of c-Src (which is associated with these receptors via adaptor proteins^*^), leading to a signaling cascade which is linked to downregulated expression of tight junction complex components (i.e., claudin-5, occludin, and zona occludens-1) and increased BBB permeability. (2) HIV-1 Tat protein enters BMECs and traffics to the nucleus (by its encoded nuclear localization signal) and upregulates expression of c-Src at the transcriptional level via direct modulation of Sp1 activity at the c-Src promotor. (3) HIV-1 Tat protein that enters BMECs may also be retained in the cytosol and directly bind the SH3 domain of c-Src, thus orchestrating the physical shift necessary to induce the active conformation of c-Src (i.e., phosphorylation at Y416 and de-phosphorylation at Y529) and leading to inhibition of BBB repair. (4) In addition, there may be super-activation of the NMDAR via a feedback loop between the ligand-binding face of NMDAR and cytosolic receptor-associated c-Src. Adaptor proteins: VRAP, VEGF receptor associated protein; TSAd, T-cell specific adaptor molecule; ND2, NADH dehydrogenase subunit 2.

## Author contributions

MM, BW, and MN all made substantial contributions to the conception or design of the work, drafted the work and revised it critically for important intellectual content, provided final approval of the version to be published, and agree to be accountable for all aspects of the work in ensuring that questions related to the accuracy or integrity of any part of the work are appropriately investigated and resolved.

## Funding

These studies were funded in part by the Public Health Service, National Institutes of Health, through grants from the National Institute of Neurological Disorders and Stroke (NS089435, MN, Principal Investigator), the National Institute of Drug Abuse (DA19807, BW, Principal Investigator), National Institute of Mental Health (MH110360, BW Principal Investigator), Comprehensive NeuroAIDS Center (CNAC) (P30 MH092177, Kamel Khalili, PI; BW, PI of the Drexel subcontract for the Clinical and Translational Research Support Core), and under the Ruth L. Kirschstein National Research Service Award T32 MH079785 (BW, Principal Investigator of the Drexel University College of Medicine component and Dr. Olimpia Meucci as Co-Director). The contents of the paper are solely the responsibility of the authors and do not necessarily represent the official views of the NIH. MN was also supported by faculty development funds provided by the Department of Microbiology and Immunology and the Institute for Molecular Medicine and Infectious Disease.

### Conflict of interest statement

The authors declare that the research was conducted in the absence of any commercial or financial relationships that could be construed as a potential conflict of interest.
